# Harnessing crayfish, *Procambarus clarkii*, to eliminate *Schistosome* transmitting snails in the Mwea irrigation scheme, Kenya

**DOI:** 10.2478/helm-2025-0020

**Published:** 2025-11-26

**Authors:** G. M. Maina, N. Mbugi, W. R. Mukabana, D. O. Odongo, E. A. Lelo

**Affiliations:** 1 Center for Biotechnology Research and Development, Kenya Medical Research Institute (KEMRI), P.O.BOX-54840-00200, Nairobi, Kenya; 2 Department of Biology, University of Nairobi Nairobi Kenya; 3 3College of Science and Technical Education, Mbeya University of Science and Technology (MUST), P.O.BOX 131, Mbeya, Tanzania

**Keywords:** Biological control, schistosomiasis, *Procambarus clarkii*, molluscivorous

## Abstract

The existence or lack of natural enemies is one ecological aspect that can have a significant impact on the human burden of environmentally transmitted parasite diseases. First discovered in Lake Naivasha, Kenya, in 1970, red swamp crayfish swiftly expanded throughout the area, overlapping with the range of freshwater snails that act as the intermediate host of schistosomiasis, a trematode disease of poverty that affects up to 250 million people worldwide, 9 million Kenyans, and 23.1 % of Mwea. While mass drug administration is practiced, cases of reinfection occur, hence the need to augment control strategies targeting snails. Using baited crayfish traps and a snail scoop, a baseline survey was carried out to map out canals with and without both the predator and the prey. Specifically, snail and crayfish populations were statistically expressed as percentages and proportions. Snail abundance and Infection rates were compared using percentages and proportions. Prior to the introduction of crayfish, in August 2021, 2703 snails that transmit schistosomes were found in the five sites in the Mwea water environment. While in the other regions, Nice had no snails, Mianya (12.3 %) had high infection rates, which were followed by Nguka (12.3 %), Murinduko (6.3 %), and Nineveh (5.0 %). In certain settings, the prevalence of snails dramatically dropped (p ≥ 0.001) following the introduction of crayfish. The five study environments’ infection rates did not differ significantly (p ≥ 0.105). Out of the 218 crayfish captured at the six study locations, Nguka had the most (104), ahead of Nice (82) and Mokou (32), indicating relative abundances of 47.7 %, 37.6 %, and 14.7 %, respectively. During the sampling period, there were no crayfish in Mianya, Murinduko, or Nineveh. Our findings imply that ecological factors like vegetation and human activity significantly influence aquatic-based biological control.

## Introduction

Tropical and subtropical areas are home to the parasite causing schistosomiasis disease (WHO, 2024). The transmission of *Schistosoma* spp. worms is inextricably linked with freshwater snails, which are primarily members of the class Gastropoda and belong to the genera *Biomphalaria, Bulinus*, and *Oncomelania* (CDC, 2023). The asexual phase of the *Schistosoma* life cycle is maintained by snails that act as intermediate hosts. They also release infectious *Schistosoma* cercariae, which are free-swimming larval worms found in lakes, ponds, and streams. When humans or ruminants bathe, wade, wash clothes, or come into contact with tainted freshwaters, these cercariae pierce their skin and infect man. Thus, schistosomiasis affects more than 250 million individuals worldwide, what results in 3.31 million disability-adjusted life years per year (Hotez *et al*., 2014).

An estimated nine million individuals are infected with schistosomiasis (SCH) in Kenya, when *Schistosoma mansoni* causes intestinal SCH, and *Schistosoma haematobium* causes urogenital SCH. Additionally, 17.5 million individuals are at risk of developing SCH (Chadeka *et al*., (2017; Kepha *et al*., 2023). A variety of symptoms, including anemia, abdominal pain, growth retardation, and diminished cognitive development in children, are caused by schistosomiasis (Gryseels *et al*., 1987), what results in more severe sickness and up to 200,000 fatalities annually worldwide (Adoka *et al*., 2014).

Schistosomiasis is nevertheless common despite its effects on human well-being and mortality, and it predominantly impacts underprivileged and marginalized populations, especially those without the public infrastructure required for access to healthcare, safe drinking water, and proper sanitation (CDC, 2023). According to estimates from the World Health Organization (WHO), 90 % of the 235 million people who were at risk of contracting schistosomiasis in 2019 and needed preventative treatment, lived in Africa (Cioli *et al*., 2014).

Although there is growing concern about selection for resistance (Chandiwana *et al*., 1991), the standard treatment, an oral dose of the anthelmintic praziquantel, is thought to be effective in disease treatment. Currently, less than half of those who require treatment (105,420,110 / 235,378,761 = 44.8 %) acquire it (Garba *et al*., 2013). It is due to high rates of re-infection caused by frequent exposure to polluted water sources (Mkoji *et al*., 1999; Sabah *et al*., 1986), or the ineffectiveness of treatment against young worms (Crandall *et al*., 2008; Sabah *et al*., 1986). Therefore, the WHO advises using mass medicine administration and snail control techniques to control and eradicate schistosomiasis, acknowledging that snail control is crucial to lowering the spread of Schistosoma spp. (McCarthy *et al*., 2006).

In the 1990s, we conducted research in the Athi River watershed that used crayfish as prey for Bulinid snails (Pengsakul *et al*., 2013). Dams, stagnant water, and benthic habitats were the main subjects of these investigations. The experiments’ findings showed that bulinid snails were totally eradicated by crayfish. The one drawback to using crayfish in makeshift water pools and dams is that during extended drought, the predators and snails both decline. However, snails can aestivate and reappear when rain returns; therefore, the predators must be restocked (Gichuki *et al*., 2019). According to the hypothesis, the number of schistosomes transmitted by snails is inversely correlated with the number of crayfish, snail predators in benthic environments (Mwai *et al*., 2021). To test this idea, we here investigate the propagation of *Procambarus clarkii* and its general impact on snails that transmit Schistosomiasis in the extensive Mwea irrigation project, which is characterized by permanent streaming water.

## Materials and Methods

### Study Site

**Fig. 1.**
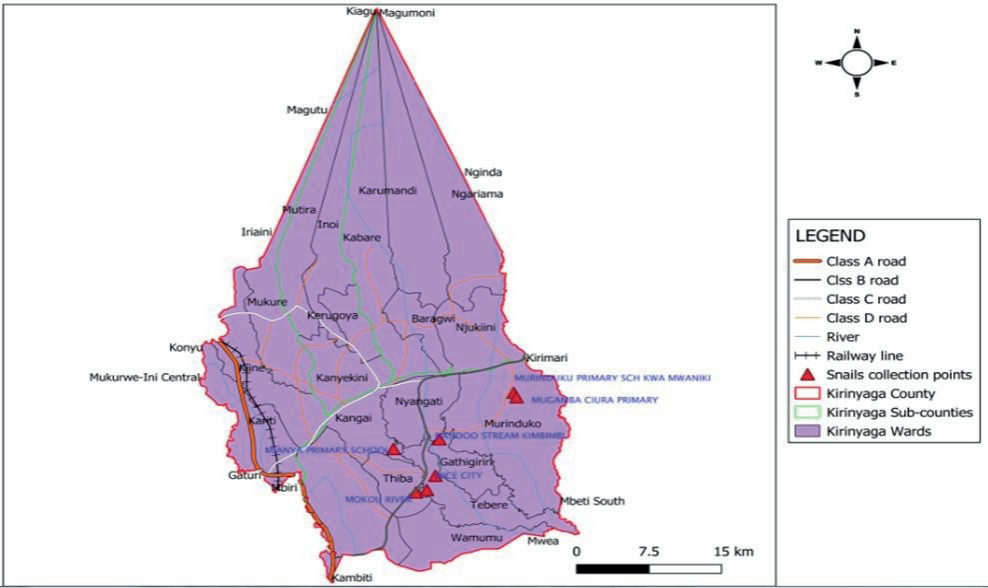
Snail collection points in Mwea Area Kirinyaga Count.

About 100 kilometers northwest of Nairobi, in the Mwea district of Kirinyaga county in central Kenya, all canals and streams were initially surveyed for the study (Fig. 1) (Allan *et al*., 2020). Kirinyaga County is located at the summit of Mount Kenya and between 1158 and 5380 meters above sea level. On average, it receives between 1200 and 1600 millimeters of rainfall annually. Based on data from the 2019 census, the county’s estimated total population was 610 411 KNBS census, 2019 (Gouvras *et al*., 2017). Since crayfish inhabit specific canals (Brown, 1994) and the sickness is very common in children and adults alike (Kibuthu *et al*., 2016), the Mwea research region was chosen for this investigation.

### Snail Sampling

Two experienced collectors scooped snails for 30 minutes per sampling site using long-handled scoops (Steel sieve with a mesh size of 2×2 mm, supported on a triangle iron frame) (Gouvras *et al*., 2017). Canal shorelines had emergent and submerged vegetation that was combed by the scoop for snails. All snails were taken to the Kimbimbi sub-county hospital lab, where they were sorted into species based on shell morphology characteristics, using standard taxonomic identification keys, counted, and screened.

### Crayfish Trapping

Three crayfish sampling and trapping techniques were employed. Meat-baited traps in water deeper than one meter were used in the initial method. Plastic 1 cm mesh was used to cover a cylindrical wire frame that was around 50 cm in length and 20 cm in diameter. The trap was submerged to remain on the bottom for a predetermined amount of time, typically two hours, after being baited with meat (Appendix 1). When the trap was lifted, *Procambarus clarkii* were manually removed via the opening after creeping in at both ends and being unable to crawl out. Snail scoops were utilized in the second trapping technique along the canals. Shallow littoral layer of emergent and sub-emergent vegetation (Appendix 2). Third, the community used ripped and frayed mosquito nets, which were dragged down the canal bed for ten minutes, then folded to capture the caught crayfish and taken out (Appendix 3).

**Appendix 1.**
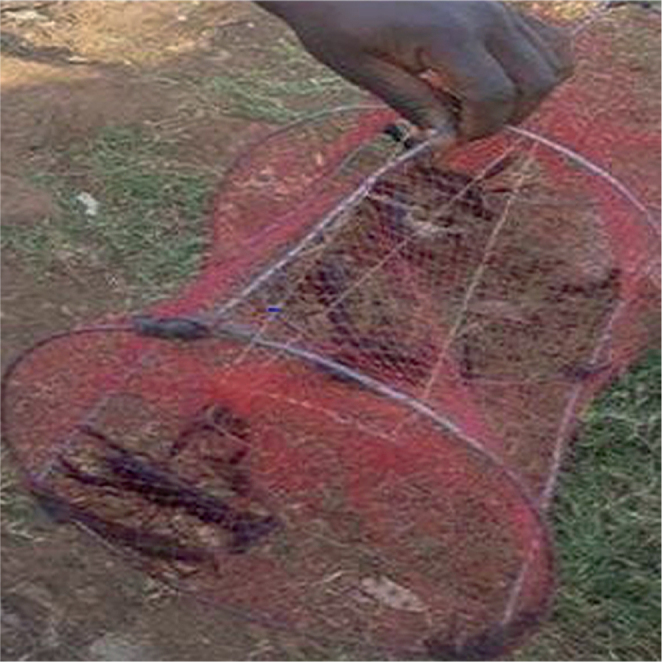
Crayfish trap with few caught *P. clarkii*.

**Appendix 2.**
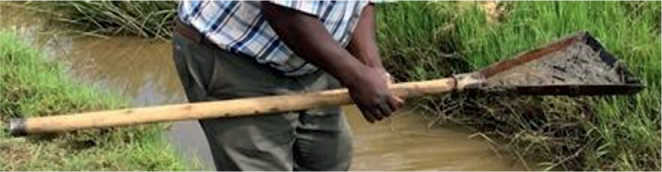
Snail scoop used to catch crayfish in littoral shallow water.

**Appendix 3.**
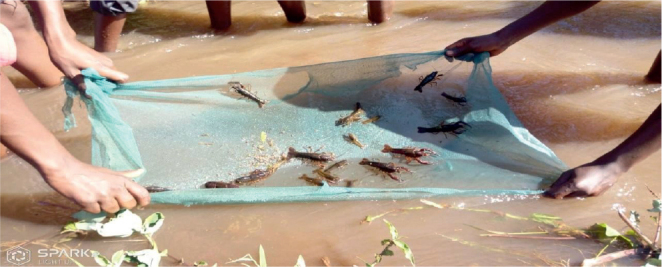
Torn and tattered mosquito net invented by villagers to trap crayfish.

### Crayfish introduction and scale-up

Six months after the baseline survey, crayfish were added to 3 of the sites where they were originally absent or in low density. The stock crayfish population originated from the Mwea canals (Fig. 1).

### Holding, Translocation, and follow-up crayfish survey

Dump gunny sacks were placed at the top and bottom of perforated buckets containing crayfish (H: 90 cm, top radius: 60 cm, bottom radius: 45 cm) for temporary confinement. Every container contained about 120 crawfish. The same day, the crayfish were transported in a covered double-cab pickup to the experimental locations. For every 100 meters of canal length, 200 crayfish were released. For the next two years, a monthly monitoring of crayfish performance and their impact on schistosome-transmitting snails was conducted. The abundance measurements, the observation of egg broods under female tails, and the appearance of juvenile and young adults were used to assess how well crayfish populations were doing.

Following stocking, crayfish samples were taken every month until the study’s conclusion. Thirty meat-baited traps were submerged in water for two hours during each sampling session. The traps were made of 2.5mm medium tensile plain flexi wire and covered with a 45 by 25 cm long nylon mesh. The simple wire is divided into multiple 45-cm pieces to build crayfish traps. The joints of the three sections are welded after they are folded into circles. At equal intervals along the three circles’ length, three straight wires were riveted at each intersection with the circular cables. At intervals of fifteen minutes, the traps were inspected. After being weighed, sexed, and tallied, the captured crayfish were sent back into the water. Because they offer the strongest indicator of crayfish abundance, the analysis concentrated on the males that were taken (Fornstrom *et al*., 1997).

### Statistical analysis

Snail and crayfish populations were expressed as percentages and proportions. Snail abundance and infection rates were compared using percentages and proportions. In addition, regression analyses were performed to determine the association between the *P. clarkii* and the snail population for all study sites.

## Ethical Approval and/or Informed Consent

The research protocol was examined and authorized by the ethical review unit (SERU) of the Kenya Medical Research Institute (Referenced; KEMRI/SERU/CBRD/PRO164/3406) and a research permit issued by the National Commission for Science and Technology, NACOSTI. (License No. NACOSTI/P/25/4175472). The study’s methodology, advantages, and possible drawbacks were explained in a sensitization presentation to the community, Mwea National Irrigation Board representatives, county health and Ministry of Education executives, and the fisheries personnel. Animals were caught, sexed, handled, and discharged under the animal welfare rules (28/1998).

## Results

### Study site selection

A total of 15 study habitats were sampled for schistosome-transmitting snails and the presence/absence of crayfish at the baseline. We settled on 5 sites for follow-up due to their ease of accessibility, financial implications, and suitability for both emergent and submerged vegetation. Follow-up was done in both dry and wet seasons between August 2021 and December 2022. GPS coordinates and abiotic parameters were recorded (Table 2).

**Table 1.** Summary of mean abiotic features over the 2-year study period and coordinates of study areas.

	Water pH	Water turbidity (ppt)	Water temp. (°C)	Water Velocity (m/S)	Air velocity (m/S)	Air temp. (°C)	GPS
**Mokou**	7.97	0.09	21.9	0.5	0.7	27.8	Lat. 0°41’5.14482” Long.37°20’40.58988”E Alt. 1115.14 M
**Nguka**	7.1	0.11	25.7	0.2	3.83	33.4	Lat. 0°38’38.55” S Long. 37°18’1772’E Alt. 1179.99M
**Nice**	7.33	0.15	25.6	0	7	29.5	Lat. 0°39’58.30”S Long. 37°21’45.38”E Alt. 1145.14M
**Mianya**	7.45	0.05	22.9	0.4	2.2	28	Lat. 0°38’10.49”S Long. 37°19’25.85’E Alt. 1168.59M
**Nineveh**	6.88	0.06	25.1	0	10.2	27.3	Lat. 0°37’31.44”S Long. 37°21’59.07”E Alt. 1145.14M

**Table 2.** Relative abundance of crayfish in the sampled sites (Baseline).

Site	*Procambarus clarkii*	Relative Abundance (%)
Mianya	0	0.0
Murinduko	0	0.0
Nice	82	37.6
Nineveh	0	0.0
Nguka	104	47.7
Mokou	32	14.7

The pH of Mokou Canal was 7.97, the highest, and the pH of the Nineveh environment was 6.88, the lowest. Fertilizer use is the primary factor that contributes to Mwea’s acidity. Perhaps as a result of its close proximity to the Nice shopping centre, Nice has the highest water turbidity, measuring 0.15 NTU (Nephelometric Turbidity Units). Since water is periodically let into the canal by activating the main canal gate valves, Mianya had the lowest turbidity, at 0.05 NTU. Mokou had the lowest average water temperature, at 21.9 °C, while Nguka had the highest, at 25.7°C. At the 0.5 m/s/S, Mokou had the fastest water speed, compared to 0 m/s in Nineveh and Nice districts.

Out of the 218 crayfish captured at the six study locations, Nguka had the most (104), ahead of Nice (82) and Mokou (32), indicating relative abundances of 47.7 %, 37.6 %, and 14.7 %, respectively. During the sampling period, there were no crayfish in Mianya, Murinduko, or Nineveh (Table 3).

**Table 3.** The association between the Crayfish and the snail population in various study sites.

Model	Site	Estimate	STD Error	R^2^	P-value	95% CI
1	Mokou	-6.002	3.165	0.3393	0.09976	-13.48561 – 1.482581
2	Mianya	-1.432	1.677	0.09435	0.4214	-5.398256 – 2.533673
3	Nineve	-2.133	3.301	0.0563	0.5387	-9.939124 – 5.672457
4	Nguka	0.8898	0.4651	0.3434	0.09729	-0.2099173 – 1.989456
5	Nice	-0.2187	0.3748	0.04639	0.5779	-1.105067 – 0.667622

### Infection rates and the abundance of crayfish

**Fig. 2.**
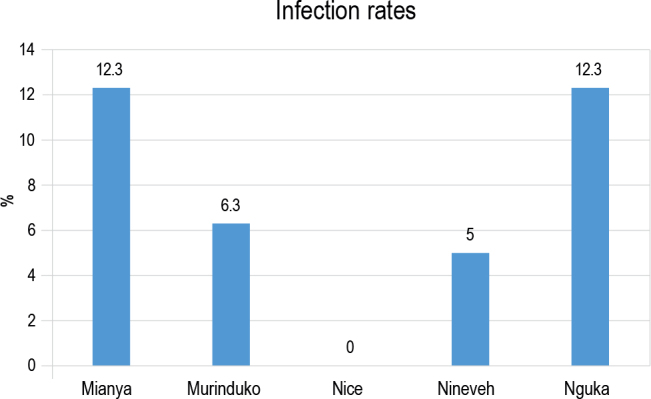
Snail abundance and prevalence of *S. mansoni* infection in the study habitats during the rainy season (November – December 2022).

To initiate shedding, snails were briefly subjected to natural light between 10:00 AM and 12:00 PM. Mianya (12.3 %) had the highest infection rates, then Nguka (12.3 %), Murinduko (6.3 %), and Nineveh (5.0 %). On the other hand, Nice Stream showed no infection rates (Fig. 2). According to the chi-squared test, all five habitats are thought to be possible places for the spread of *S. mansoni* infections. The five study environments’ infection rates did not differ significantly (p = 0.105).

Snails were much less common (p=0.001) during the research period following the emergence of crayfish, especially in Nice and Mianya. A collection of 306 crayfish was sampled on the research locations during the initial follow-up visit. Except for Nineveh and Murinduko, all study locations saw overall crayfish survival. Water velocity (p=0.014) and vegetation cover (p≥0.026) were substantially correlated with crayfish abundance. It’s interesting to note that, in comparison to the other locations, we found the most crayfish and the fewest snails in the Nice stream (Fig. 4).

Mianya canal, located beside a primary school, had almost 10 % of the 95 *B. pfeifferi* collected shedding *S. mansoni*. Nineveh Canal had the fewest snails, both infected and uninfected (Fig. 2).

**Fig. 3.**
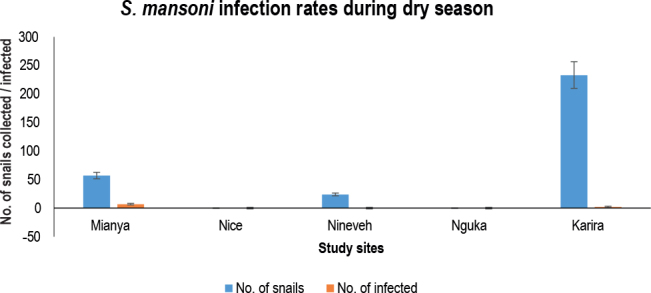
Snail abundance and prevalence of *S. mansoni* infection in the study habitats during the dry season (March – April 2022).

Since there was no water in the Nguka and Nice habitats during the dry season, there were no snails (Fig. 3). Commercial structures were gradually encroaching on the study area in the Nice environment. There were 50 *B. pfeifferi* snails and a few shedders in the Mianya environment. To supply nearby rice crops, irrigation water flowed throughout the ecosystem, encompassing the Karira habitat, which was not included in the study, and produced the most significant number of snails.

**Fig. 4.**
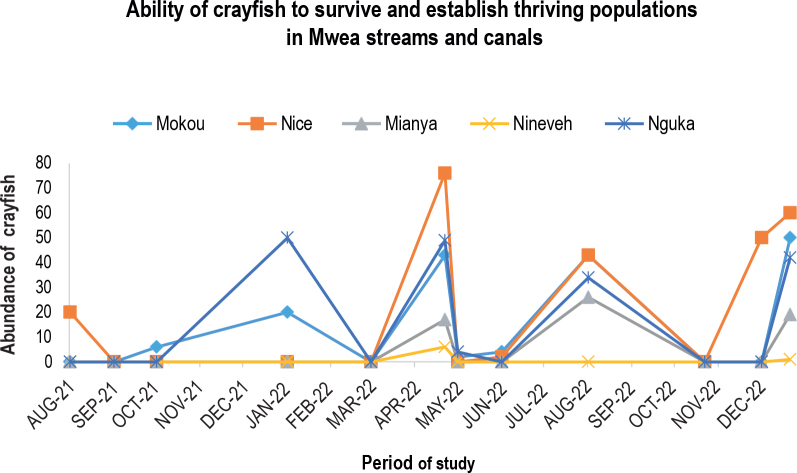
Ability of crayfish to survive and establish thriving populations in Mwea streams habitats after introduction.

The January 2022 sampling time point saw a peak of crayfish in the Mokou environment. In August 2021, there were a lot of crayfish in the nice habitat, and in April 2022, they peaked. Slow-moving water, minimal habitat disturbance, and favorable reed and hedge grass conditions were all present in this area. Few crayfish were found in the fast-moving canal that is Mianya’s habitat, most likely as a result of the water’s quick flow. There were also a few crayfish in Nineveh. December 2021, January 2022, and April 2022 saw three significant surges in the Nguka habitat (Fig. 4).

**Fig. 5.**
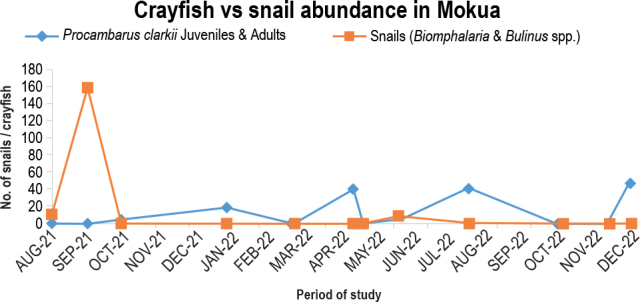
Predation potential of *P. clarkii* in Mokou stream.

Habitats experience seasonal changes in predator/prey relationships based on biotic and abiotic factors. After the crayfish introduction was completed in August 2021, there were twelve snails in the area. In September 2021, the snail population increased to 161. However, between October 2021 and January 2022, the crayfish successfully suppressed snail numbers by briefly establishing themselves. Crayfish numbers steadily decreased from March to December 2022, presumably as a result of the canal gate valves opening and letting a lot of water flow, which makes it impossible for crayfish to survive. In the littoral zones, snails can cling to plants; a snail population surge was noted in June 2022, but the snails were exterminated in December 2022 (Fig. 5).

**Fig. 6.**
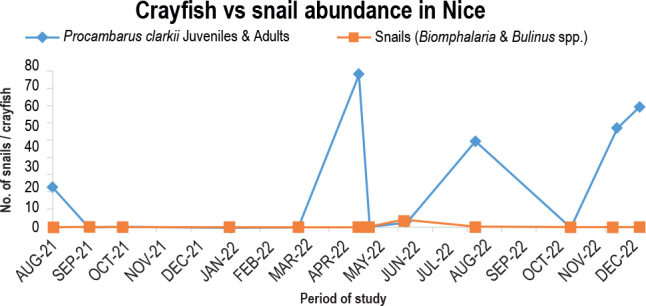
Predation potential of *P. clarkii* in Nice stream.

For the August 2021 sampling point, the Nice Stream near the Nice shopping complex had 20 crayfish and zero snails. The sampling point at 3 snails in June 2022 saw the sole high. The snails were eradicated mainly by crayfish, which peaked at 50 predators in the December 2022 survey point (Fig. 6).

**Fig. 7.**
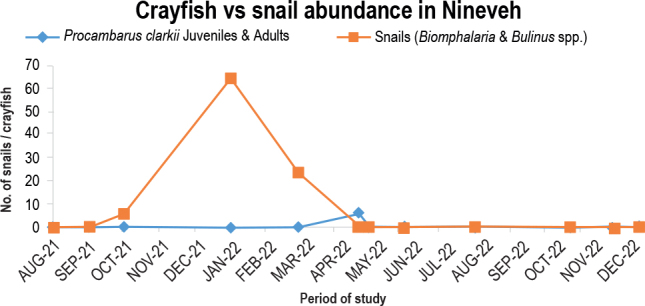
Predation potential of *P. clarkii* in Nineveh stream.

The number of snails in Nineveh, a habitat near the Kimbimbi hospital, peaked in January 2022 at 64. In December 2021 and April 2022, a small colony of crayfish with one and six predators, respectively, was discovered at this research site. Due to a lack of water in the canal and a decrease in vegetation cover brought on by human activity, no snails were captured between April and December 2022 (Fig. 7).

**Fig. 8.**
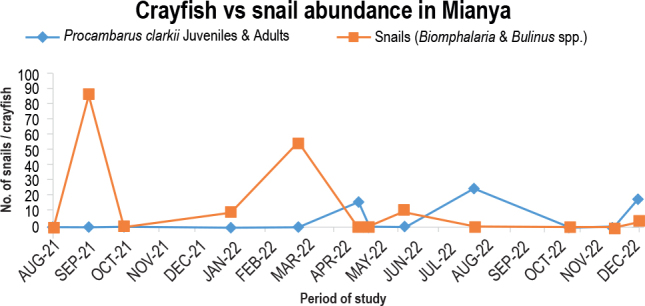
Predation potential of *P. clarkii* in Mianya stream.

Using 19, 17, and 26 predators, respectively, the crayfish population was sampled in December 2021 and again in April and August 2022. The snail population, however, remained stable between September 2021 and March 2022. Snail populations peaked at 88 in September 2021 and 57 in March 2022, respectively (Fig. 8). The predator population suppressed snails during the survey period; just 29 snails were sampled in September 2021. The crayfish population, on the other hand, peaked four times in December 2021, January, April, and August 2022, with 42, 50, 49, and 34 predators, respectively. The population was nil from August to October 2021.

Water levels remained relatively constant, with only slight variations, throughout the research period, except in May 2022, when rains followed a minor rise in water levels. All study habitats, irrespective of habitat type, held water. Except for April and June, when it varied between 25 and 28°C, the water temperature remained relatively constant throughout the observation period, ranging between 22 and 24°C.

The studied environments supported a diverse range of vegetation cover over the year, including papyrus, rice grass (weed), arrow roots, and grass. Both seasons of the year had different densities of vegetation cover, which corresponded to the times when rice was grown in the study environments. During rice regeneration, there was always vegetation covering. Nevertheless, at the first harvest in December, while the other farmers altogether collected the rice, all study locations had water, which helped to sustain the snail populations. Harvesting, clearing vegetation, and ploughing and levelling the rice fields were among the other human endeavors frequently seen during the current investigation.

In several study sites, abiotic factors such as the fast water flow amid both short and long-term rains were shown to affect the crayfish’s capacity to survive. For example, the Nineveh stream had a year-round fast water flow of up to 0.83 m/s. Furthermore, the stream’s lack of foliage covers leads to a number of human endeavors in the vicinity, such as washing motorbikes.

We found conflicting results here because of the heavy pesticide application in the paddies next to this stream and due to the higher water velocity (Gaynor *et al*., 2018; Venter *et al*., 2016). The snail population would decline shortly after the introduction of crayfish. Crayfish would progressively decrease as Biomphalaria snails increased after the rice cultivation, fertilizer application, and increasing water volume. Nevertheless, only in Nice and Mianya, where crayfish persisted, was this association noted. High densities of flora, including papyrus and arrow roots, were also present at the study sites. Crayfish abundance was substantially correlated with both the density of vegetation and water velocity (p≥0.007) (Fig. 10). In addition, regression analyses were conducted to unveil the existing association between the *P.clakii* and the snail’s intermediate host of schistosomiasis for all study sites. It was demonstrated that there were a negative association between the *P. clakii* and snails in all study sites, except Nguka. However, the association was not statistically significant at p-value ≥0.05 (Table 3).

**Fig. 9.**
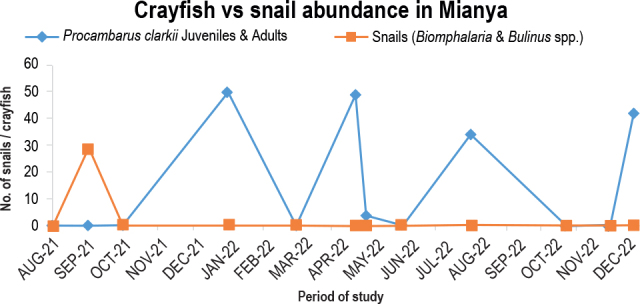
Predation potential of *P clarkii* in Nguka stream.

**Fig. 10.**
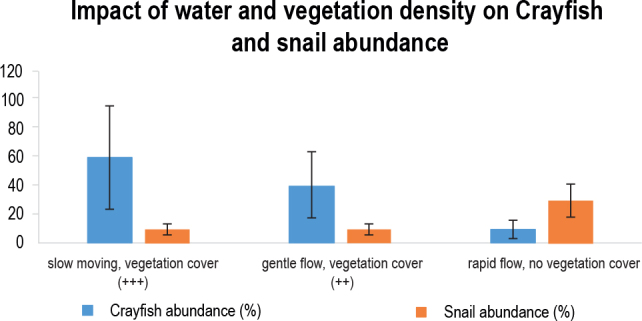
Impact of water velocity and vegetation cover on predation potential of crayfish against snails.

A checklist of some of the fauna and flora found within the study area. Numbers of species was not taken into account across the study habitats. The most common emergent vegetation was arrow arum (Moore *et al*., 2016). The plant forms optimum breeding conditions for both crayfish and snails. (Table 4)

**Table 4.** Species Checklist of some of the native aquatic biodiversity.

Taxa	Scooped, trapped and seen
Crustaceans	Crabs and crayfish
Coleoptera	Water beetles
Hemiptera	Water scorpions, Belostoma
Ephemeroptera	Mayfly
Odonata	dragonfly
Plecoptera	stonefly
Hirudina	Leeches
Pisces	Cichlids, mudfish
Diptera	Adult mosquitoes and larva
Ceratopogonidae	biting midges
Gastropoda	Bi-valves, Lymnea, Belamya, Pila ovata, Melanoides tuberculata, Ancylids, physa acuta, Apple snail eggs and adults, Ceratophallus.
Nematodes	Oligochaetes,
Water fowls	Comorants, Little egrets, Egyptian geese, open billed stocks, whistling ducks, quilea quilea, African jacana
Amphibians	Toads and frogs
Reptiles	Monitor lizards *(Varanus niloticus)*
Macrophytes	**Submerged**: *Ceratophyllum demersum* and *Najas horrida*. **Emergent:** Arrow arum, Papyrus, **Floating aquatic plants:** Water lettuce, Elephant’s eye, Water hyacinth

## Discussion

Increased transmission of schistosomiasis to humans is linked to irrigation schemes because they increase the volume of surface water that snail intermediate hosts can infest. Therefore, in many tropical and subtropical regions of the world, the implementation of irrigation schemes meant to boost agricultural output has had the unintended effect of introducing or increasing schistosomiasis transmission (Cox *et al*., 2022; Smith *et al*., 2019). In the Mwea irrigation project, this study sought to examine how crayfish reduce schistosome-transmitting snails and how biotic and abiotic factors affect predator-prey interactions.

Following the introduction, the number of crayfish in the stream rose and then somewhat decreased in September 2022. Whereas the number of crayfish increased over time and gradually stabilized as the research length passed, the number of snails in the stream was large at the beginning, then decreased and ultimately petered out in May 2022. This finding is consistent with our earlier research in Kenya’s Machakos County, in which snail populations sharply declined in regions where crayfish had established themselves in lentic setups (Smith *et al*., 2020). Snails can be consumed by crayfish as they develop their colony. In addition, the observed negative associations in the regression analysis further reiterate the potential of the crayfish to reduce the vector population. Nevertheless, the colonization of crayfish was also threatened by human factors.

Further, water pollution was seen in the Nineveh stream as a result of numerous human activities, including washing cars and motorcycles. These elements cause snails to reappear and crayfish to diminish rapidly. The effectiveness of this approach depends on striking a balance between biocontrol measures and human factors. Because there were little human activities in the Nice habitat, the crayfish population remained stable. This finding was in line with earlier research. Anthropogenic disruptions affect the composition of wildlife communities and animal behaviour on almost 75 % of the Earth’s terrestrial area (Cebrián *et al*., (1992; Desouky *et al*., 2013; Morolli *et al*., 2006).

From August 2021 to December 2022, human activities frequently cleared stream bank macrophytes in the Nineveh environment (Fig. 7), interfering with predator refugia and causing maladaptation. Each is motivated to maximize fitness in the setting of their surroundings and the other’s geographical and temporal niche by the predator-prey response race (Stara *et al*., 2016; Yu *et al*.,2017). *Procambarus clarkii*, the red swamp crayfish, is a common freshwater crayfish and an important species in many bodies of water. Ecosystems, including the food web, biodiversity, sediment, and water quality, are all greatly impacted (Bubb *et al*., 2006; Perez-Saez *et al*., 2019a; Robinson *et al*., 2000). In addition to its important role in malacophagy, *P. clarkii* has been employed extensively as a model organism in the assessment of pollution and water quality (Gámez-Virués *et al*., 2015; Landis *et al*., 2008; Tscharntke *et al*., 2005).

Ephemeral home ranges are adopted by crayfish in streams, where they forage in one refuge area for a few days before relocating to another (Meehan *et al*., 2011; Scherr *et al*., 2008). Particularly in the Mianya stream, wherein the relative abundance increased from 10.4 to 15.4 months and 1 year after crayfish release, respectively, our results are consistent with those of Bubb and Robinson *et al*. Our findings, nevertheless, contradict those of Bub *et al*. in Nineveh, where the crayfish were unable to properly acclimate, establish, and disperse.

In contrast to earlier research, which concentrated on lentic and lotic ecosystems, this study was distinct (Bommarco *et al*., 2013). However, by utilizing research by Perez-Alvarez *et al*. (Staudacher *et al*., 2018), This work is modelled on augmentative biological control by strengthening native natural enemies, the crayfish, in an intensive agricultural irrigation setting.

One of the primary causes of the decline of biodiversity and the simplified nature of the landscape is agricultural intensification (Figiel *et al*., 1991). By reducing crop species diversity and converting natural habitats into additional farmed areas, intensive agriculture methods simplify the landscape. Arthropod species diversity and composition are directly impacted by those shifts in land-use patterns (Lefcort *et al*., 1993), and they may also lessen the provision of vital ecosystem services such as biological pest control (Davis *et al*., 2015). As a result, agricultural systems have relied more and more on artificial inputs, which worsens the harm that intensified agriculture causes to both biodiversity and environmental preservation (Gherardi *et al*., 2001). Under these circumstances, it is imperative to support farming methods that balance agricultural output with biodiversity protection and sustainable use (Chandler *et al*., 2016). Their degree of control varied considerably over the growing season and between landscapes, despite our findings suggesting that naturally generated enemies can help regulate *B. pfeifferi* populations. Consequently, to achieve reliable and cost-effective snail control, complementary techniques are preferred. Our study’s findings imply that, in some ecological settings, augmentative deployments of predators may be able to enhance the effectiveness of naturally occurring crayfishes in controlling snails.

Throughout our investigation, snail predation was constantly greater at predator-supplemented sites than in control plots. However, due to variations in the makeup of the environment, predator augmentation was unable to manage inherent planorbids across sites consistently. The efficacy of augmentative biological control in the field may be limited by several ecological phenomena, as documented by earlier research (e.g., climate restrictions, release timing, rates, and quality control). This study aims to highlight the importance of landscape context in undermining the efficacy of enemy augmentation as a vector management tactic.

Numerous non-mutually exclusive mechanisms could account for the landscape-moderated effectiveness of predator enhancement on pest control. The antagonistic interactions (i.e., intraguild predation and predator disruption) between enemy species in simple landscapes (March *et al*., 2006), functional complementarity between augmented and resident enemies across complicated landscapes (Wäckers, 2003), and changes in the local enemy assemblage composition mediated by the landscape, might subsequently determine the sign and strength of connections with the augmented predators (Landis *et al*., 2000).

First, the intensity of vector inhibition can be increased by increasing the complementarity between resident and augmented opponents due to the complexity of the landscape (Winkler *et al*., 2003). Complicated environments may favor the simultaneous existence of species with overlapping feeding niches with a high proportion of semi-natural habitats, which might offer natural competitors with adequate microhabitats and alternate food sources (McCarthy *et al*., 2006).

Depending on the kind of predator involved, the impact of aquatic vegetation and complex habitats on predator-prey interactions can vary (Hayes *et al*., 2012; Marston *et al*., 2016; Martín *et al*., 2001). Crayfish typically use a sit-and-wait tactic, which enables them to catch prey successfully in a variety of settings (Carlsson *et al*., 2009). Nevertheless, our findings contradict An earlier investigation by Chandler *et al*. (2016) that found that crayfish are similarly effective predators in wetland environments with varying amounts of herbaceous plants. Because the Nice Canal habitat featured denser foliage cover over the other four habitats, and human interference in the form of riverbank removal, the crayfish were able to flourish and destroy B. pfeifferi (Fig. 7). The results of March *et al*. (2006), who discovered that native forest areas had more than twice as many freshwater crayfish burrows as in other land uses, align with our results.

### Increasing the success of biological control

Specific biological control strategies have a likelihood of being effective, but they need extra human inputs either prior to discharge, through the crop environment’s natural colonization, or to promote post-release establishment. This is particularly the case with conservation biological control. Growers have been urged to plant specific nectar-producing flowers within field boundaries to make the crop environment more appealing to these natural enemies, such as adult parasitoids and hoverflies, which need sources of pollen and nectar to mature their eggs (Maldonado *et al*., 2019). There is also some experimental proof suggesting adding flowers to the crop may enhance the organic control of pests (Perez-Saez *et al*., 2019a; Sacchi *et al*., 2021).

Our findings provide credence to the hypothesis that, in complex environments where habitat variety may foster favorable conditions for compatibility between enhanced and resident enemies, boosting enemy numbers may have net positive impacts on vector control as well as health status (Bonds *et al*., 2010).

A native freshwater snail of the Río de la Plata basin in South America, the apple snail *Pomacea canaliculata* (Ampullariidae) (Parker, 1992; Tanner, 1989). This apple snail is regarded as a highly effective invasive species (Carlsson *et al*., 2009), which has spread feral populations across the globe, particularly in East and Southeast Asia. It has additionally caused significant ecological damage to wetlands and a significant loss of revenue in aquatic crops. In line with previous research by Djeddour *et al*. (2021), we found that this ampullariid snail invaded rice, canals, and both terrestrial and aquatic plants through our field surveys and sampling in the Mwea irrigation scheme.

According to a study conducted in Argentina by Maldonado *et al*., the presence of the apple snail significantly decreased the fertility of Biomphalaria peregrina and *Physa acuta* (Chandiwana *et al*., 1991; Cioli *et al*., 2014). It is unknown from our current investigation how the apple snail affects the effectiveness of planorbid snail repression and near extinction. It is worthwhile to investigate if the near extinction of *B. pfeifferi* was caused by crayfish alone or by a combination of crayfish and apple snail.

### Study limitations

Several potential limitations and confounders in our experimental approach deserve consideration. The appearance of an exotic snail species, *Pomacea canaliculata*, may have played a role as an additive biological control (Maldonado *et al*., 2019). Second, crayfish are primarily nocturnal (Sacchi *et al*., 2021). Our crayfish sampling was done during the day, so our catch may not represent a fair and accurate abundance of the predators in the studied habitats. Third, this study was conducted during the COVID-19 pandemic, when lockdowns and the NTD program’s travel restrictions were suspended. Scooping is a labor-intensive exercise, and wearing masks makes it more challenging. Although quadrat snail sampling provides an absolute number of snails (Perez-Saez *et al*., 2019), the study habitat was primarily covered by cattail grass (Typha angustifolia) and green arrow Arum (Peltandra virginica), rendering quadrat sampling infeasible. Consequently, a timebased sampling technique was employed.

### Proposed future studies and recommendations

Since crayfish is an exotic invasive species (Albrecht *et al*., 2025), future studies should explore The use of sex hormones to generate mono-sex predators for release in Schistosomiasis endemic regions.

## Conclusion

Our results encourage more studies involving individuals in biological control initiatives for long-term sustainability after financing expires. Neighborhoods would also be crucial to conservation biological management, which is modifying the environment to improve the longevity, fertility, behavior, and survival of natural enemies. By improving their third trophic level (natural enemies), habitat management can decrease the abundance of vectors. Conversely, bottom-up influences can suppress vectors by strengthening the first trophic level. (flora, as observed at Nice habitat) of various habitats.

With the arrival of the invasive apple snail in the Mwea irrigation scheme, further studies are needed to elucidate the impact of this ampullariid snail on *B. pfeifferi* and the interaction of crayfish and the apple snail on the ecosystem. It will be interesting to know the origin of the invasive snail in Mwea, whether it was anthropogenically translocated, and why. Lastly, crayfish, though an undisputed snail predator, face challenges from farm agrochemicals, which should be prudently applied to realize the full control potential of this predator.
